# Adiponectin may contribute to synovitis and joint destruction in rheumatoid arthritis by stimulating vascular endothelial growth factor, matrix metalloproteinase-1, and matrix metalloproteinase-13 expression in fibroblast-like synoviocytes more than proinflammatory mediators

**DOI:** 10.1186/ar2844

**Published:** 2009-11-02

**Authors:** Hyun-Mi Choi, Yeon-Ah Lee, Sang-Hoon Lee, Seung-Jae Hong, Dae-Hyun Hahm, Sang-Yun Choi, Hyung-In Yang, Myung Chul Yoo, Kyoung Soo Kim

**Affiliations:** 1 East-West Bone & Joint Research Institute, East-West Neo Medical Center, Kyung Hee University, 149 Sangil-dong, Gangdong-gu, Seoul, 134-727, Republic of Korea; 2 Division of Rheumatology, Department of Internal Medicine, College of Medicine, Kyung Hee University, Hoegi-1-dong, Dongdaemun-gu, Seoul, 130-702, Republic of Korea; 3 Division of Rheumatology, Department of Internal Medicine, East-West Neo Medical Center, Kyung Hee University, 149 Sangil-dong, Gangdong-gu, Seoul, 134-727, Republic of Korea; 4 Acupuncture and Meridian Science Research Center, Kyung Hee University, Hoeggi-dong, Dongdaemoon-gu, Seoul, 130-702, Republic of Korea; 5 School of Life Sciences and Biotechnology, Anam-dong Seongbuk-gu, Korea University, Seoul, 136-701, Republic of Korea

## Abstract

**Introduction:**

The role of adiponectin in the pathogenesis of arthritis is still controversial. This study was performed to examine whether adiponectin is involved in joint inflammation and destruction in rheumatoid arthritis (RA) in relation to the expression of vascular endothelial growth factor (VEGF) and matrix metalloproteinases (MMPs).

**Methods:**

Synovial cells from RA patients were treated with adiponectin or interleukin (IL)-1β for 24 hours. The culture supernatant was collected and analyzed for the levels of IL-6, IL-8, prostaglandin E_2 _(PGE_2_), VEGF, and MMPs by enzyme-linked immunosorbent assay. The levels of adiponectin, VEGF, MMP-1, and MMP-13 in the joint fluids from 30 RA or osteoarthritis (OA) patients were also measured.

**Results:**

Adiponectin at the concentration of 10 μg/mL stimulated the production of IL-6, IL-8, and PGE_2 _in RA fibroblast-like synoviocytes (FLSs), although the level of these was much lower than with 1 ng/mL IL-1β. However, adiponectin stimulated the production of VEGF, MMP-1, and MMP-13 at the same level as IL-1β. In addition, the level of adiponectin and MMP-1 in the joint fluid of RA patients was significantly higher than in OA patients. Adiponectin was positively correlated with VEGF in RA patients but not in OA patients, while the level of MMPs in joint fluid was not correlated with adiponectin in either RA or OA patients.

**Conclusions:**

Adiponectin may play a significant role in the pathogenesis of RA by stimulating the production of VEGF and MMPs in FLSs, leading to joint inflammation and destruction, respectively.

## Introduction

Adipose tissue, once viewed as simply a storage and release depot for lipids, is now considered an endocrine tissue [1,2] that secretes various substances (adipokines), including tumor necrosis factor-alpha (TNF-α), interleukin (IL)-6, leptin, adiponectin, resistin, visfatin, and omenetin [3,4]. Among these adipokines, much attention has been paid to adiponectin's relationship with insulin sensitivity and glucose and lipid metabolism in the past 10 years. In addition, adiponectin is known to exhibit potent anti-inflammatory [5], atheroprotective [6], and antidiabetic [7] effects.

Recent findings suggest that adiponectin may be involved in the pathogenesis of rheumatoid arthritis (RA). Levels of adiponectin in synovial fluid and sera were elevated in patients with RA [8,9]. Adiponectin also induces the production of proinflammatory cytokines, IL-6, matrix metalloproteinase (MMP)-1, and IL-8 from RA synovial fibroblasts *in vitro *[10,11]. Thus, it was suggested that adiponectin can also exert significant proinflammatory and matrix-degrading effects. However, the role of adiponectin in the pathogenesis of RA is still controversial because of conflicting reports about its function [10,12-15]. In particular, adiponectin seems to play an anti-inflammatory role because it significantly inhibited IL-1β-stimulated synovial cell proliferation in collagen-induced arthritic mice, despite increased IL-6 expression [16]. In contrast, high-grade inflammation in RA patients was negatively correlated with circulating adiponectin concentrations [17]. Rather, it was suggested that circulating adiponectin may be involved in cardiovascular disease in RA patients. Although this contradiction was partly explained by the induction of tolerance to inflammatory stimuli by adiponectin [18], the pro- or anti-inflammatory effects of adiponectin on the pathogenesis of RA remain unknown.

With regard to adiponectin's proinflammatory effects, we wondered whether adiponectin might stimulate the production of vascular endothelial growth factor (VEGF) and MMPs as well as proinflammatory mediators like IL-1β and TNF-α do. In this study, we investigated the stimulatory effect of adiponectin on the production of IL-6, IL-8, prostaglandin E_2 _(PGE_2_), VEGF, and MMPs. In addition, the correlation between adiponectin and VEGF or MMPs was investigated by measuring the levels of these three proteins in the joint fluid of patients with RA or osteoarthritis (OA).

## Materials and methods

### Cell culture

All *in vitro *experiments were carried out with fibroblast-like synoviocytes (FLSs) derived from patients with RA. After informed consent was obtained, synovial tissues were collected from RA patients. They met the 1987 American College of Rheumatology criteria for the diagnosis of RA and had been treated with nonbiological disease-modifying antirheumatic drugs and had undergone therapeutic joint surgery. FLSs were isolated and grown in Dulbecco's modified essential medium (low glucose) (Gibco-BRL, now part of Invitrogen Corporation, Carlsbad, CA, USA) supplemented with 10% (vol/vol) fetal bovine serum (Invitrogen Corporation) and 1× Antibiotic-Antimycotic (Invitrogen Corporation) as described previously [19]. After the cells had grown to confluence, they were split at a 1:4 ratio. FLS passages 3 to 6 from three patients were used for all experiments. Ethical permission was obtained from the Institutional Review Board for Human Research of Kyung Hee University, Neo-Medical Center.

### Measurement of gene expression by enzyme-linked immunosorbent assay

Synovial cells (2.5 × 10^5 ^cells per 60-mm dish per 2 mL of serum-free media) were treated with recombinant adiponectin (1 or 10 μg/mL) or IL-1β (0.1 or 1 ng/mL) (ProSpec, Rehovot, Israel). Conditioned media was collected 24 hours later. Briefly, FLS cultures were centrifuged and the supernatants were collected and analyzed for IL-6, IL-8, PGE_2_, VEGF, MMP-1, and MMP-13 with an enzyme-linked immunosorbent assay (ELISA) kit (R&D Systems, Inc., Minneapolis, MN, USA). Three independent experiments were performed in quadruplicate. Each experiment was performed using synovial cells from different patients. For the assessment of MMP-1, MMP-13, VEGF, and adiponectin levels in joint fluid, the collected joint fluid from 30 patients with RA or OA was dispensed into 1-mL aliquots and treated with hyaluronadase at 50 μg/mL for 1 hour at room temperature. The joint fluid was diluted with diluent's buffer for the proper detection range with ELISA. The levels of proteins of interest in joint fluid were measured using a commercial ELISA kit from R&D Systems, Inc., as described above.

### Real-time polymerase chain reaction

Real-time quantitative polymerase chain reaction (PCR) was carried out using the LightCycler PCR system (Roche Diagnostics, Meylan, France) using the DNA binding SYBR Green I dye to detect the PCR products as described previously [19]. A serial dilution was used to generate each standard curve. Each 20-μL reaction mixture contained 1× LightCycler-DNA master SYBR Green I, a specific primer, and 2 μL of cDNA. After 2 minutes of denaturation at 95°C, the MMPs, VEGF, IL-6, IL-8, cyclooxygenase-2 (COX-2), and β-actin underwent 40 reaction cycles at 95°C for 5 seconds, 55°C to 60°C for 10 seconds (annealing), and 72°C for 13 seconds. Product specificity was determined by melting curve analysis as described in the LightCycler manual. The results are calculated as ratios of gene transcripts to β-actin transcripts, with the quantity of transcripts in each sample expressed as a copy number. The ratio of MMPs, VEGF, IL-6, IL-8, and COX-2/β-actin mRNA was assigned a value of 100%, with the corresponding results calculated as relative percentages. The primers were synthesized by Bioneer Co. Ltd. (Seoul, Korea), and their sequences are listed in Table 1.

**Table 1:** The sequence of polymerase chain reaction primers used in this experiment

Primer name	Primer sequence	Product size
MMP-1 sense	5'-CCT AGC TAC ACC TTC AGT GG-3'	338 bp
MMP-1 antisense	5'-GCC CAG TAC TTA TTC CCT TT-3'	
MMP-13 sense	5'-TTG AGG ATA CAG GCA AGA CT-3'	311 bp
MMP-13 antisense	5'-TGG AAG TAT TAC CCC AAA TG-3'	
VEGF sense	5'-ACT TCA GGC TCT TCT CCT TT-3'	288 bp
VEGF antisense	5'-TTC AGA CAA CCT GAG TCC TT-3'	
IL-6 sense	5'-CTG GTC TTT TGG AGT TTG AG-3'	344 bp
IL-6 antisense	5'-TTT CTG ACC AGA AGA AGG AA-3'	
IL-8 sense	5'-ACT TTC AGA GAC AGC AGA GC-3'	264 bp
IL-8 antisense	5'-GTG GTC CAC TCT CAA TCA CT-3'	
COX-2 sense	5'-TTC AAA TGA GAT TGT GGG AAA ATT GCT-3'	306 bp
COX-2 antisense	5'-AGA TCA TCT CTG CCT GAG TAT CTT-3'	
β-actin sense	5'-TCA TGA GGT AGT CAG TCA GG-3'	305 bp
β-actin antisense	5'-CTT CTA CAA TGA GCT GCG TG-3'	

bp, base pairs; COX-2, cyclooxygenase-2; IL, interleukin; MMP, matrix metalloproteinase; VEGF, vascular endothelial growth factor.

### Western blot analysis

FLSs cultured (2.5 × 10^5 ^cells) in 60-mm dishes were serum-starved overnight and stimulated by adiponectin (1 or 10 μg/mL) or IL-1β (0.1 or 1 ng/mL) for 24 hours. The cells were subsequently washed twice in phosphate-buffered saline and treated with 50 μL of lysis buffer (20 mM Tris-Cl [pH 8.0], 150 mM NaCl, 1 mM EDTA [ethylenediaminetetraacetic acid], 1% Triton X-100, 20 μg/mL chymostatin, 2 mM PMSF [phenylmethylsulphonyl fluoride], 10 μM leupeptin, and 1 mM AEBSF [4-(2-aminoethyl)benzenesulfonyl fluoride]). The samples were separated using 12% SDS-PAGE and were then transferred to Hybond-ECL [enhanced chemiluminescence] membranes (Amersham, now part of GE Healthcare, Little Chalfont, Buckinghamshire, UK). The membranes were first blocked with 6% nonfat milk dissolved in TBST buffer (10 mM Tris-Cl [pH 8.0], 150 mM NaCl, 0.05% Tween 20). The blots were then probed with various rabbit polyclonal antibodies for COX-2 and β-actin (Cell Signaling Technology, Inc., Danvers, MA, USA) diluted 1:1,000 in Tris-buffered saline at 4°C overnight and incubated with 1:1,000 dilutions of goat anti-rabbit IgG secondary antibody coupled with horseradish peroxidase. The blots were developed using the ECL method (GE Healthcare). For re-probing, the blots were incubated in the stripping buffer (100 mM 2-mercaptoethanol, 2% SDS, 62.5 mM Tris-HCl [pH 6.7]) at 50°C for 30 minutes with occasional agitation.

### Statistical analysis

The *in vitro *experimental data are expressed as the mean ± standard error of the mean of quadruplicate samples. Differences between groups were assessed by repeated analysis of variance followed by the Dunnett multiple comparison test. The level of MMPs, VEGF, and adiponectin in the joint fluid of RA and OA patients was compared between groups with the unpaired *t *test. To determine the degree of linearity between two variables, data were compared using the Pearson correlation test (two-tailed). Prism software 4 (GraphPad Software, Inc., San Diego, CA, USA) was used for statistical analysis and graphing. Differences were considered significant at a *P *value of less than 0.05.

## Results

### Effect of adiponectin on the production of proinflammatory mediators in rheumatoid arthritis fibroblast-like synoviocytes

**Figure 1:**
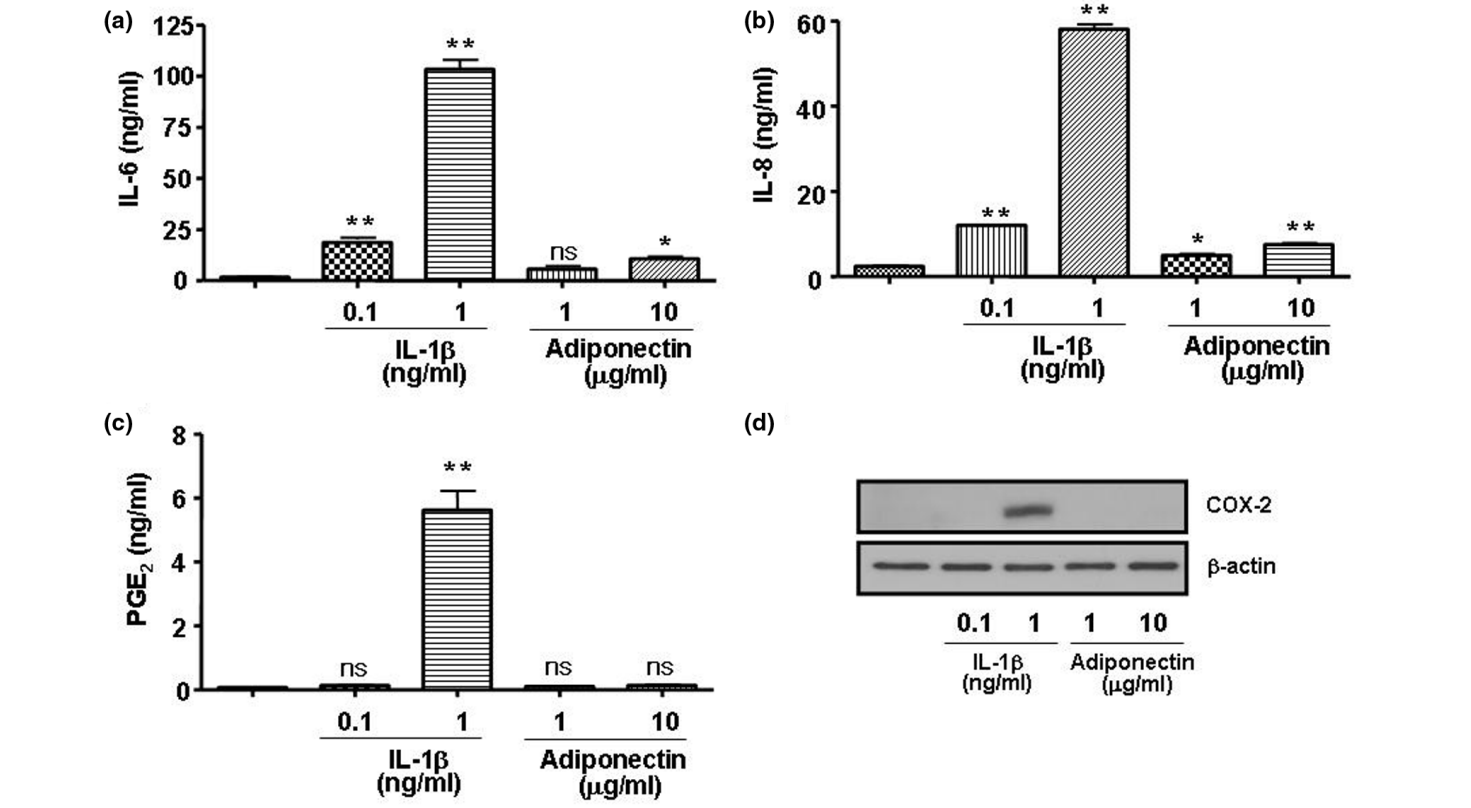
Comparative effect of adiponectin and interleukin (IL)-1β on the production of IL-6, IL-8, and prostaglandin E_2 _(PGE_2_) in rheumatoid arthritis fibroblast-like synoviocytes. IL-1β at 1 ng/mL stimulates the production of **(a) **IL-6, **(b) **IL-8, and **(c) **PGE_2 _more than 10 μg/mL adiponectin. **(d) **The expression of cycloxygenase-2 (COX-2) increased only in the presence of IL-1β in proportion to the level of PGE_2_. The data shown are representative of three independent experiments, and similar results were obtained from all three. Values are expressed as mean ± standard error of the mean. * *P *< 0.05, ** *P *< 0.01 versus nontreated group. ns, not significant.

To evaluate whether adiponectin contributes significantly to the inflammation of joints in RA patients, the level of proinflammatory mediators in response to IL-1β or adiponectin was compared in FLSs from patients with RA (Figure 1). The RA synovial cells were treated with 1 or 10 μg/mL adiponectin. For comparison, the cells were treated with 0.1 or 1 ng/mL IL-1β. Adiponectin stimulated the production of IL-6 and IL-8 in RA FLSs, but the production of these proteins was very low in comparison with that induced by IL-1β in these cells. IL-1β at 1 ng/mL increased the production of IL-6 and IL-8 about fivefold and ninefold, respectively, compared with adiponectin at 10 μg/mL. In particular, 1 ng/mL IL-1β increased the level of PGE_2 _about 40-fold more than IL-1β at 0.1 ng/mL and adiponectin at 10 μg/mL. Consistent with the protein levels, the mRNA levels of the respective genes were also increased by the evaluation of real-time PCR (data not shown). This result suggests that adiponectin may not greatly induce the production of proinflammatory mediators such as IL-6, IL-8, and PGE_2 _in FLSs like IL-1β does.

### Effect of adiponectin on the production of vascular endothelial growth factor and matrix metalloproteinases in rheumatoid arthritis fibroblast-like synoviocytes

**Figure 2:**
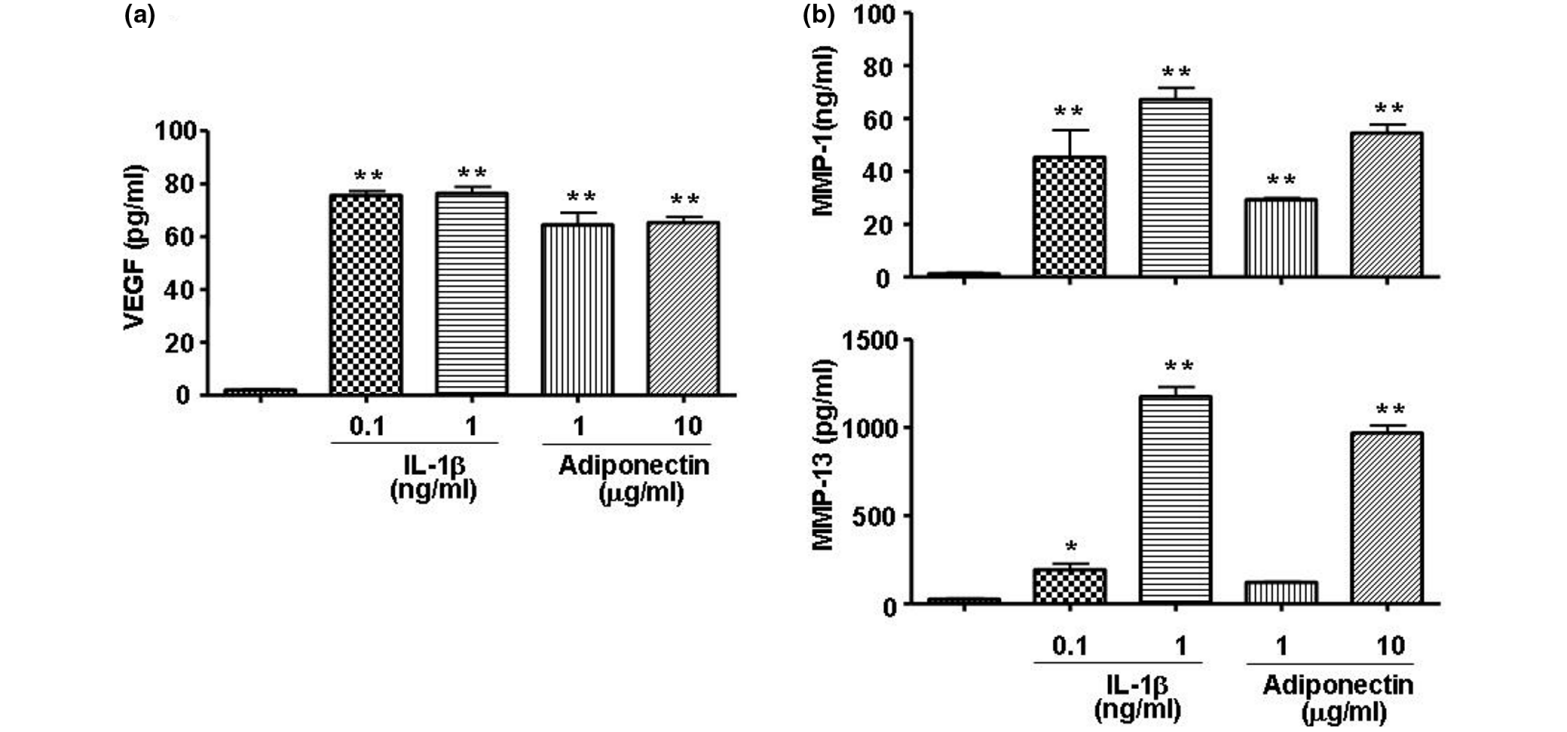
Effect of adiponectin on the expression of vascular endothelial growth factor (VEGF), matrix metalloproteinase (MMP)-1, and MMP-13 in rheumatoid arthritis fibroblast-like synoviocytes. Adiponectin stimulates the expression of **(a) **VEGF and **(b) **MMP-1 and MMP-13 at a level comparable to 1 ng/mL interleukin-1-beta (IL-1β). The data shown are representative of three independent experiments, and similar results were obtained from all three. Values are expressed as mean ± standard error of the mean. * *P *< 0.05, ** *P *< 0.01 versus nontreated group.

Next, to evaluate whether adiponectin stimulates the production of VEGF and MMPs for angiogenesis of pannus and joint destruction in RA FLSs, VEGF and MMP production was evaluated in the supernatants of cell cultures treated with adiponectin or IL-1β (Figure 2). Both adiponectin and IL-1β strongly stimulated the production of VEGF, MMP-1, and MMP-13 in RA FLSs. However, the expressions of MMP-2 and MMP-9 were not increased by either adiponectin or IL-1β at the mRNA or protein level. Consistent with the mRNA levels (data not shown), the protein levels of VEGF, MMP-1, and MMP-13 elevated by treatment with 10 μg/mL adiponectin were similar to those after treatment with 1 ng/mL IL-1β. The difference between adiponectin and IL-1β was not statistically significant, suggesting that adiponetin may have a role in the production of VEGF and MMPs like IL-1β does.

### Correlation of matrix metalloproteinase levels with adiponectin in the joint fluid of patients

**Figure 3:**
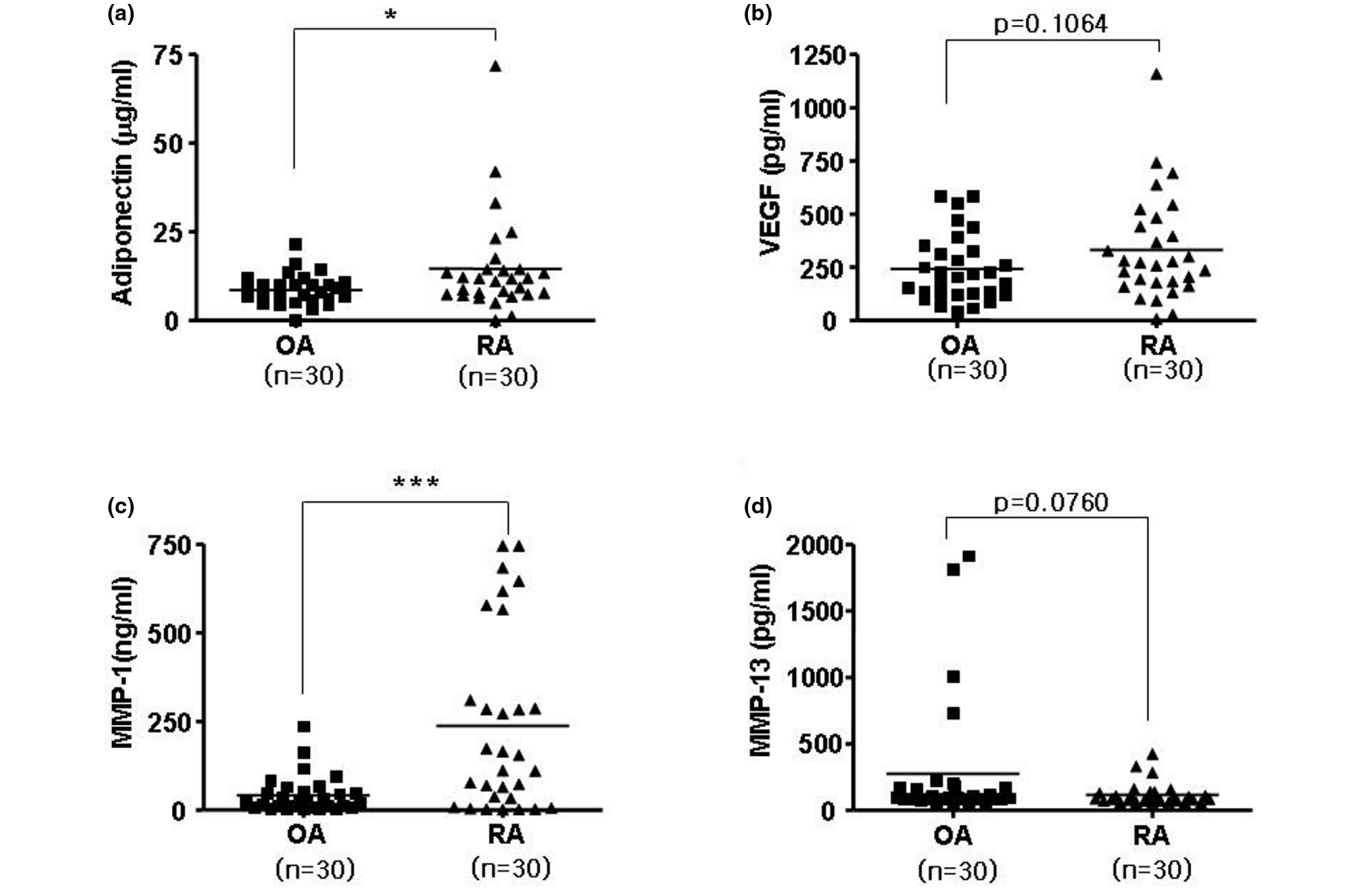
The levels of adiponectin, vascular endothelial growth factor (VEGF), matrix metalloproteinase (MMP)-1, and MMP-13 in the joint fluid of patients with rheumatoid arthritis (RA) or osteoarthritis (OA). The joint fluid levels of **(a) **adiponectin and **(c) **MMP-1 were significantly higher in RA patients than in OA patients, whereas **(b) **VEGF and **(d) **MMP-13 were not statistically different between RA and OA patients. Joint fluid samples from 30 patients in each group were used for the analysis. Values are expressed as mean ± standard error of the mean. * *P *< 0.05, *** *P *< 0.001 versus OA patient group.

**Figure 4:**
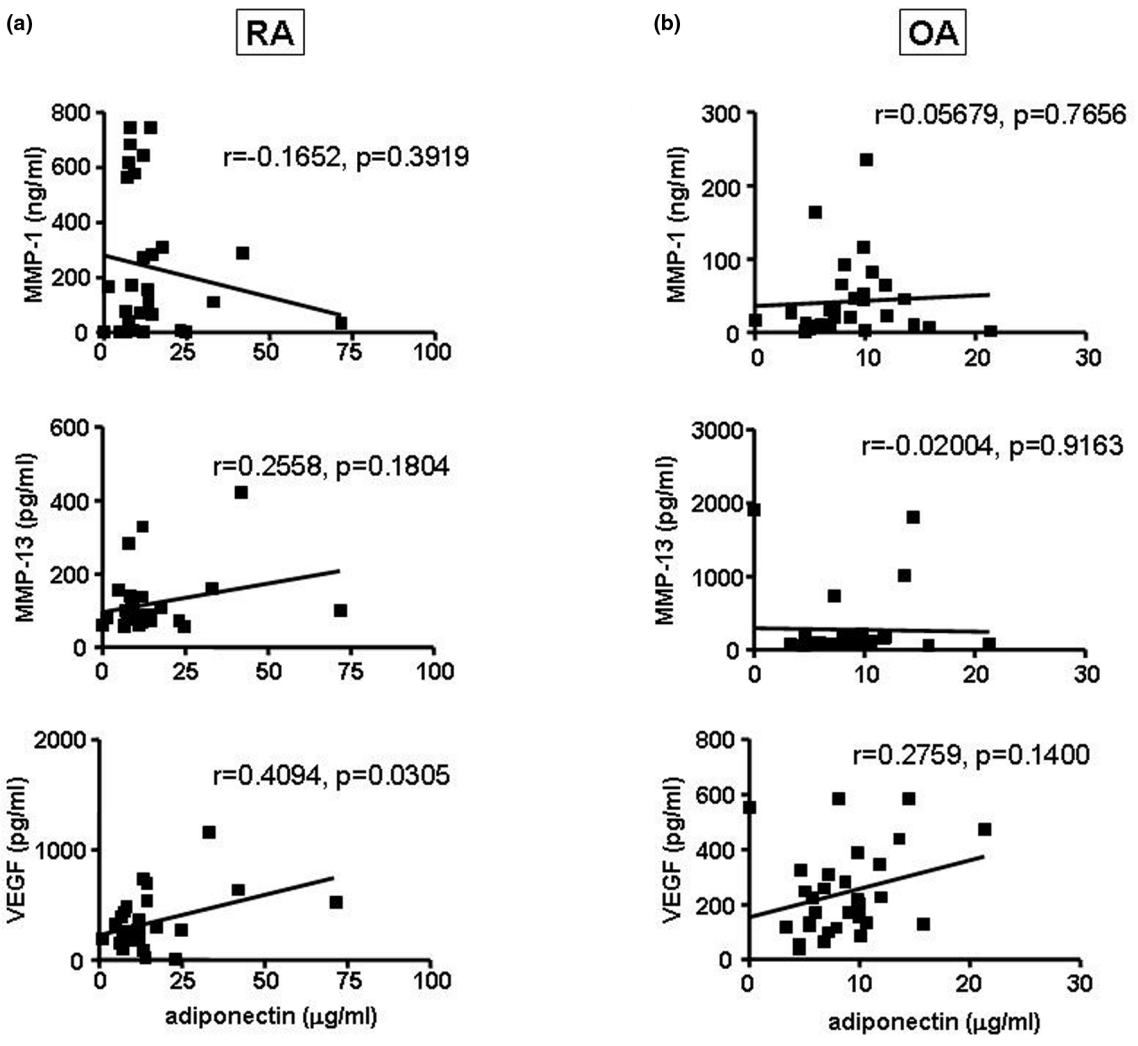
Correlation of adiponectin with vascular endothelial growth factor (VEGF), matrix metalloproteinase (MMP)-1, or MMP-13 in the joint fluid from rheumatoid arthritis (RA) or osteoarthritis (OA) patients. The level of adiponectin was positively correlated with the level of VEGF in the joint fluid of **(a) **RA patients, but not **(b) **OA patients. The level of adiponectin did not correlate with the levels of MMP-1 and MMP-13 in either RA or OA patients.

To evaluate whether the *in vitro *effect of adiponectin on the expression of VEGF, MMP-1, and MMP-13 is associated with the expression of these genes in the joint fluid of RA patients, we measured the levels of adiponectin, VEGF, MMP-1, and MMP-13 in the joint fluid of RA or OA patients (Figure 3). As reported previously [8,9], the level of adiponectin was significantly elevated in the joint fluid of RA patients compared with that of OA patients (Figure 3a). The MMP-1 level was also higher in RA patients than in OA patients (Figure 3b). However, the level of MMP-13 and VEGF was not significantly different between RA and OA patients (Figure 3c, d). Next, we checked whether adiponectin levels correlated with VEGF, MMP-1, and MMP-13 levels in the joint fluid from RA or OA patients (Figure 4). However, the level of adiponectin did not positively correlate with MMP levels in the joint fluid of either RA or OA patients. MMP levels in joint fluid might be affected by other factors like proinflammatory cytokines and hypoxia or by unknown factors. However, the adiponectin levels significantly and positively correlated with VEGF levels in RA joint fluid, but not OA joint fluid. This result suggests that adipopectin may play a role in inducing the expression of VEGF in RA FLSs, leading to angiogenesis and the formation of pannus in RA patients.

## Discussion

Adiponectin is known to play a significant role in the pathogenesis of RA, although whether adiponectin acts as an anti-inflammatory or proinflammatory mediator is controversial. Assuming that adiponectin plays a role in the pathogenesis of arthritis as a proinflammatory mediator, we tried to determine how significantly adiponectin contributes to joint inflammation and destruction compared with another proinflammatory cytokine, IL-1β. Thus, we evaluated the effect of adiponectin on the production of the proinflammatory mediators VEGF and MMPs and compared the result with that of IL-1β, one of the major proinflammatory cytokines in RA FLSs.

In this study, RA FLSs stimulated with 10 μg/mL adiponectin increased the production of IL-6, IL-8, and PGE_2_. However, the level of these proteins was low in comparison with that of RA FLSs stimulated with 1 ng/mL IL-1β. Adiponectin also greatly increased the mRNA and protein levels of collagenases (MMP-1 and MMP-13) and VEGF. MMPs and VEGF were elevated to a level comparable to that of IL-1β in RA FLSs. Interestingly, the mRNA and protein levels of gelatinases (MMP-2 and MMP-9) were not stimulated by either adiponectin or IL-1β. These results are consistent with some, but not all, previous results. Ehling and colleagues [10] reported that adiponectin increased the expression of pro-MMP-1 and IL-6 in RA FLSs, but not pro-MMP-13. In addition, adiponectin stimulated the expression of MMP-9, but not MMP-2, in cultured murine chondrocytes [20].

**Figure 5:**
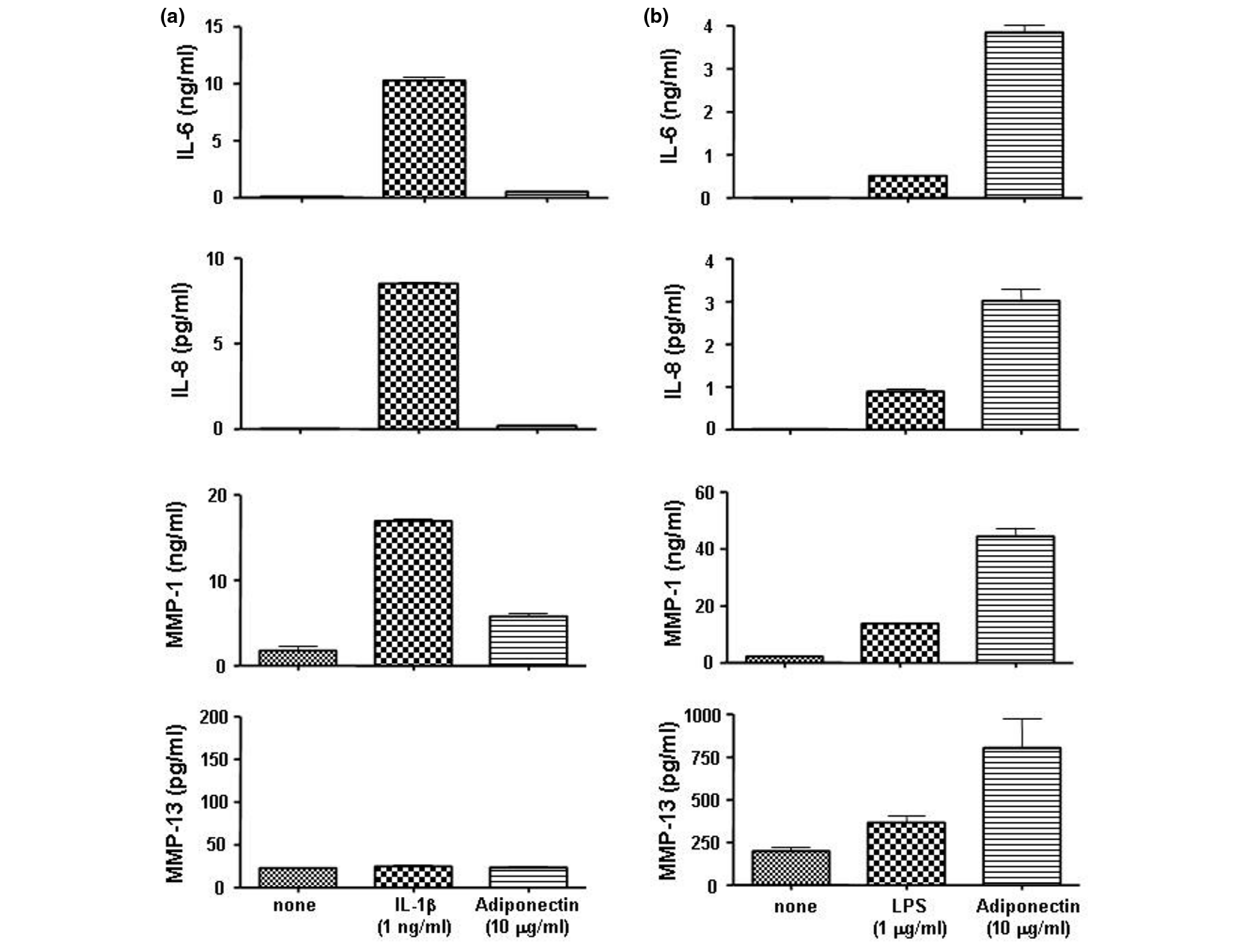
Testing possible adiponectin-mediated factors that may stimulate matrix metalloproteinase (MMP) expression in rheumatoid arthritis (RA) fibroblast-like synoviocytes (FLSs). **(a) **Adiponectin-mediated interleukin (IL)-6 and IL-8 may stimulate MMP expression, but the levels of MMPs at 6 hours of stimulation are very low compared with that stimulated by IL-1β, which did not stimulate MMP expression in RA FLSs. **(b) **Recombinant adiponectin protein contaminated with lipopolysacchride (LPS) could stimulate MMP expression; however, LPS at 1 μg/mL did not strongly stimulate the expression of these proteins compared with adiponectin at 10 μg/mL. The data shown are representative of two independent experiments, which were performed in duplicate. Similar results were obtained from the two experiments.

Some questions about this effect may be raised. First, the human recombinant adiponectin protein used in these studies is a single nonglycosylated polypeptide chain produced in *Escherichia coli *with a molecular weight of 25.1 kDa and containing 231 amino acids. Thus, the adiponectin used in this system may not completely duplicate the effect of native glycosylated adiponectin under physiological conditions. However, the adiponectin monomer forms multimeric complexes under physiological conditions and include a low-molecular-weight trimeric form (75 to 90 kDa) made up of three monomers strongly bound together at the globular domain level, a medium-molecular-weight hexameric form, and a high-molecular-weight form (about 500 kDa) [21]. The biological activity of each form has not yet been established. Furthermore, a proteolytic cleavage product of adiponectin, known as globular adiponectin, circulates in human plasma [22]. The percentage of each form with respect to the total amount of adiponectin could vary as a function of the different pathophysiological conditions. Thus, the biological activities may differ according to the different isoforms of adiponectin. However, we are confident that this result may represent, at least in part, the effect of a physiological adiponectin. Second, the concentration of adiponectin used to treat cells may have been at a physiological concentration. However, the level of IL-1β (1 ng/mL) used in this study may not represent the physiological concentration given that its concentration in the joint fluid of patients with RA is about 200 pg/mL [23]. Thus, a comparison of *in vitro *effects of adiponectin and IL-1β may not represent the *in vivo *effect of these factors. Adiponectin may play a more important role in the stimulation of MMPs in FLSs than IL-1β does. Our study provides insight into an understanding of adiponectin's role in joint destruction and inflammation in RA patients. Third, the cells were stimulated with adiponectin for 24 hours. Since potent stimulatory factors such as IL-6 and IL-8 are induced by adiponectin soon after stimulation with this factor, the effects observed with adiponectin may be mediated by these proinflammatory cytokines. However, we evaluated the levels of IL-6 and IL-8 after 6 hours of stimulation with adiponectin or IL-1β. The production levels of IL-6 and IL-8 after treatment with adiponectin were about 400 and 200 pg/mL, respectively; such levels are too low to increase MMP expression in this system. In contrast, IL-1β stimulated IL-6 and IL-8 protein expression to levels of about 10 and 8 ng/mL, respectively. This result suggests that adiponectin-mediated proinflammatory cytokines IL-6 and IL-8 could not increase the production of MMPs (Figure 5a). Finally, a recombinant protein produced in an *E. coli *system may have endotoxin contamination, and the endotoxin contaminant could stimulate the production of MMPs and VEGF. It was reported that commercial recombinant adiponectin contained lipopolysacchride (LPS) at concentrations of up to 30 pg/μg of protein [18]. However, even at LPS concentrations of 1 μg/mL, the production of MMPs, IL-6, and IL-8 was only weakly stimulated and the levels of these proteins were much lower than after treatment with 10 μg/mL adiponentin (Figure 5b). Therefore, we believe that the increase in MMPs and VEGF in this system was due to the effect of adiponectin alone.

Next, we determined whether adiponectin affected the gene expression levels of the joint fluid in patients with arthritis *in vitro *by measuring the levels of adiponectin, MMPs, and VEGF in the joint fluid of patients with RA or OA, and we checked for a correlation between adiponectin and MMPs or VEGF. The level of adiponectin was not correlated with MMP levels in the joint fluid of either RA or OA patients. However, adiponectin was positively correlated with VEGF in the joint fluid of RA patients. Although the synovial adiponectin level was significantly higher in RA patients than in OA patients in our study (a result that is consistent with a previous report [13]), the elevated adiponectin may counteract the local inflammatory process since the adiponectin was negatively associated with local inflammatory factors in patients with RA. In addition, the level of adiponectin and VEGF in the joint fluids of RA or OA patients did not correlate with the level of C-reactive protein, suggesting that adiponectin may not be significantly involved in inflammation (data not shown). Furthermore, it was suggested that adiponectin may have a protective role in OA [14]. Nevertheless, serum adiponectin concentrations correlate with the severity of RA evaluated by the extent of joint destruction, indirectly suggesting that adiponectin may be involved in joint destruction by stimulating the production of MMPs [24]. In contrast, VEGF levels were positively correlated with adiponectin levels in RA joint fluid, but not in OA joint fluid, although a correlation does not necessarily imply a causal relationship given that the levels may be affected by various factors. Thus, we cannot explain why there are differences between the two groups, even though the VEGF level between the two groups was not significantly different. The relationship between adiponectin and VEGF has not been addressed previously. Inconsistent with our data, treatment with recombinant adiponectin in a mouse model of laser-induced choroidal neovascularization resulted in decreased levels of VEGF [25]. Also, adiponectin did not regulate VEGF release in human airway smooth muscle cells whereas leptin did stimulate VEGF release [26]. However, the concentration used in these treatments may not be the physiological concentrations. In our *in vitro *experiments, 1 to 10 μg/mL adiponectin was used to detect increased gene expression of various factors. At adiponectin concentrations of less than 1 μg/mL, the genes in RA FLSs were not activated. If its physiological concentration is taken into consideration, adiponectin should be used at a concentration of at least 1 μg/mL for *in vitro *experiments [13].

Pathological processes cannot be fully understood based on the change in expression of individual genes alone since various factors act in concert in the development of specific diseases in the body. Thus, systems biology can provide a novel conceptual framework for understanding a disease [27]. Joint inflammation in arthritic patients is a complex immune reaction that is affected by various factors. Thus, joint inflammation should be understood as the integrated results of several factors, such as immune cell types infiltrating the joint cavity, cytokines, adipokines, hypoxia, and so on. In our study, the *in vitro *effect of adiponectin on MMP production was not demonstrated in the joint fluid of RA patients by evaluating the correlation between MMPs and adiponectin. The expression of MMPs also seems to be differentially regulated by various factors, including hypoxia (unpublished data) and nuclear factor-kappa-B inhibitors [28,29]. In addition, MMP levels may be more significantly affected by the stage of disease than other factors, such as cytokines. MMP-9 level was increased more in early stages of arthritis than in later stages [30]. Thus, the level of MMPs in the joint fluid of RA patients may not be affected by IL-1β or adiponectin alone. Rather, an integral effect of various unknown factors may impact the level of MMPs in joint fluid.

Factors that increase the expression of MMPs and VEGF have been suggested as potential therapeutic targets to delay or reduce the joint destruction that occurs in RA patients [31]. Based on the *in vitro *effect of adiponectin on the expression of MMPs and VEGF, adiponectin may be a potential target to block MMP or VEGF expression. However, further studies should be performed to better understand factors that control the expression of MMPs and VEGF in the joint fluid of RA patients. This knowledge may open new doors to treatment and prevent the pathological processes of RA.

## Conclusions

We show for the first time that adiponectin increases the level of VEGF and MMPs in RA FLSs as much as IL-1β but causes much smaller increases in IL-6, IL-8, and COX-2 compared with IL-1β. Furthermore, the level of adiponectin in the joint fluid of RA patients, but not OA patients, positively correlated with the level of VEGF. Although these data suggest a role for adiponectin in the perpetuation of synovitis in RA, no conclusions can be drawn from these results with regard to the relationship between adiponectin and inflammatory arthritis. Additional mechanistic and longitudinal studies in humans in the future might help to correlate our findings with clinical features.

## Abbreviations

COX-2: cyclooxygenase-2; ECL: enhanced chemiluminescence; ELISA: enzyme-linked immunosorbent assay; FLS: fibroblast-like synoviocyte; IL: interleukin; LPS: lipopolysacchride; MMP: matrix metalloproteinase; OA: osteoarthritis; PCR: polymerase chain reaction; PGE_2_: prostaglandin E_2_; RA: rheumatoid arthritis; TNF-α: tumor necrosis factor-alpha; VEGF: vascular endothelial growth factor.

## Competing interests

The authors declare that they have no competing interests.

## Authors' contributions

KSK and MCY participated in the data analysis and the design of the study and drafted the manuscript. HMC, YAL, and SHL performed the experiments. SJH and HIY provided the synovium and joint fluids from patients and participated in the design of the study. All authors read and approved the final manuscript.
